# Stimulation-Dependent Intraspinal Microtubules and Synaptic Failure in Alzheimer's Disease: A Review

**DOI:** 10.1155/2012/519682

**Published:** 2012-03-08

**Authors:** Fuyuki Mitsuyama, Yoshio Futatsugi, Masato Okuya, Tsukasa Kawase, Kostadin Karagiozov, Yoko Kato, Tetsuo Kanno, Hirotoshi Sano, Shizuko Nagao, Tadashi Koide

**Affiliations:** ^1^Department of Neurosurgery, Fujita Health University, 1-98, Kutsukake, Toyoake, Aichi 470-1101, Japan; ^2^Department of Pediatric Neurology, Tomishiro Central Hospital, 25, Azaueda, Tomishiro, Okinawa 901-0243, Japan; ^3^Department of Internal Medicine, Tenjyu Hospital, 138, Komegase, Kita, Nagoya, Aichi 462-0031, Japan; ^4^Education and Research Center of Animal Models of Human Diseases, Fujita Health University, 1-98, Kutsukake, Toyoake, Aichi 470-1101, Japan; ^5^Fukuyu Medical Institute, 75-1, Asahigaoka, Meito, Nagoya, Aichi 465-0041, Japan

## Abstract

There are many microtubules in axons and dendritic shafts, but it has been thought that there were fewer microtubules in spines. Recently, there have been four reports that observed the intraspinal microtubules. Because microtubules originate from the centrosome, these four reports strongly suggest a stimulation-dependent connection between the nucleus and the stimulated postsynaptic membrane by microtubules. In contrast, several pieces of evidence suggest that spine elongation may be caused by the polymerization of intraspinal microtubules. This structural mechanism for spine elongation suggests, conversely, that the synapse loss or spine loss observed in Alzheimer's disease may be caused by the depolymerization of intraspinal microtubules. Based on this evidence, it is suggested that the impairment of intraspinal microtubules may cause spinal structural change and block the translocation of plasticity-related molecules between the stimulated postsynaptic membranes and the nucleus, resulting in the cognitive deficits of Alzheimer's disease.

## 1. Introduction

What is the precise mechanism of memory disturbance in patients with Alzheimer's disease (AD)? For the mechanism of memory storage in the normal brain, it is commonly believed that a long-lasting change in synaptic function is the cellular basis of learning and memory [1–3], especially at the hippocampal Schaffer collateral synapses on CA1 pyramidal cells. The best characterized form of such synaptic plasticity is the long-term potentiation (LTP), which is observed at excitatory synapses in the CA1 region of the hippocampus [4–6]. These synapses are spatially distributed on the dendrites of CA1 pyramidal neurons. A remarkable feature of LTP is that a short period of tetanic stimulation can initiate a persistent increase in synaptic transmission lasting several hours and often longer. 

 There were two remarkable discoveries in the 1990s that examined memory storage.

One discovery is that some signal translocation to the stimulated postsynaptic membrane (an anterograde transport) is essential for inducing LTP in CA1 neurons, including AMPA receptors [7–11], CaMKII [12–14]. The other discovery is that the expression of late stages of LTP requires protein synthesis and gene expression [15, 16]. The requirement for transcription during LTP indicates that signals generated at the synapse must be transmitted to the nucleus for LTP induction. Although these bidirectional transports of signals are believed to be essential to induce LTP, their precise motor mechanism is not known.

 The second discovery described above was an important step toward understanding the mechanisms of memory storage, but the specificity of plasticity at stimulated synapses, as occurs in CA1 neurons, may require other mechanisms in addition to an increase in the transcription level. To explain this synaptic specificity, the synaptic tagging theory proposes that a tag is activated in activated synapses, and this localizes the effects of plasticity-promoting molecules that otherwise travel nonspecifically in the neuron [17–20]. This synaptic tagging theory implicitly acknowledges the importance of synaptic specificity in memory storage.

**Figure 1:**
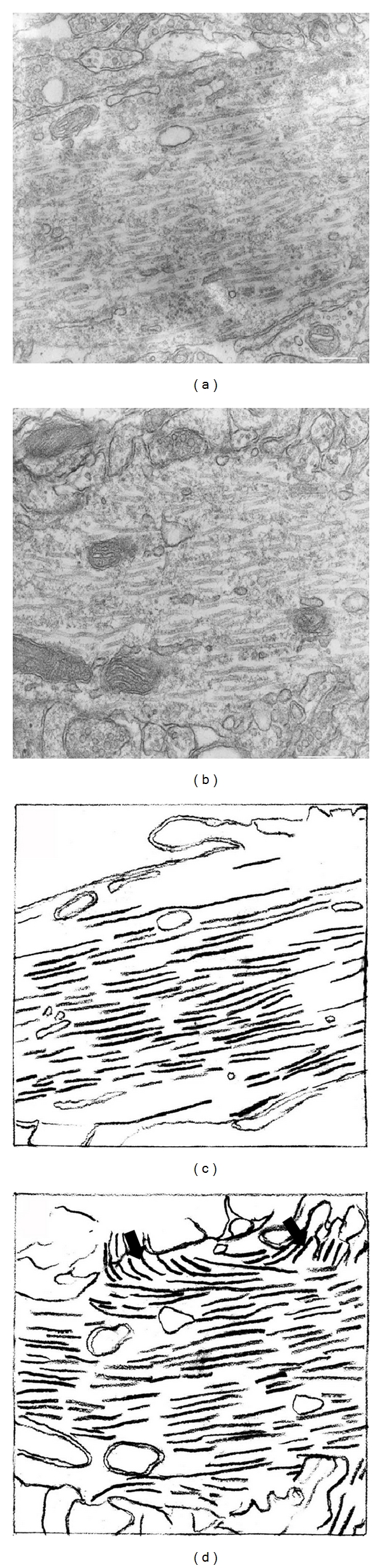
Redistribution of microtubules in dendritic shafts of hippocampal CA1 neurons after tetanic stimulation during LTP. A relatively small dendrite (about 1200 nm in diameter) of CA1 neurons in a nonstimulated hippocampal slice (a) and (c). Similar size of dendrite of CA1 neurons in a stimulated hippocampal slice (b) and (d). Many subcortical microtubules which went in the direction of spines emerged after stimulation (arrows in (d)). Reprinted from Mitsuyama et al. (2008) [21] with permission. Bar, 200 nm.

**Figure 2:**
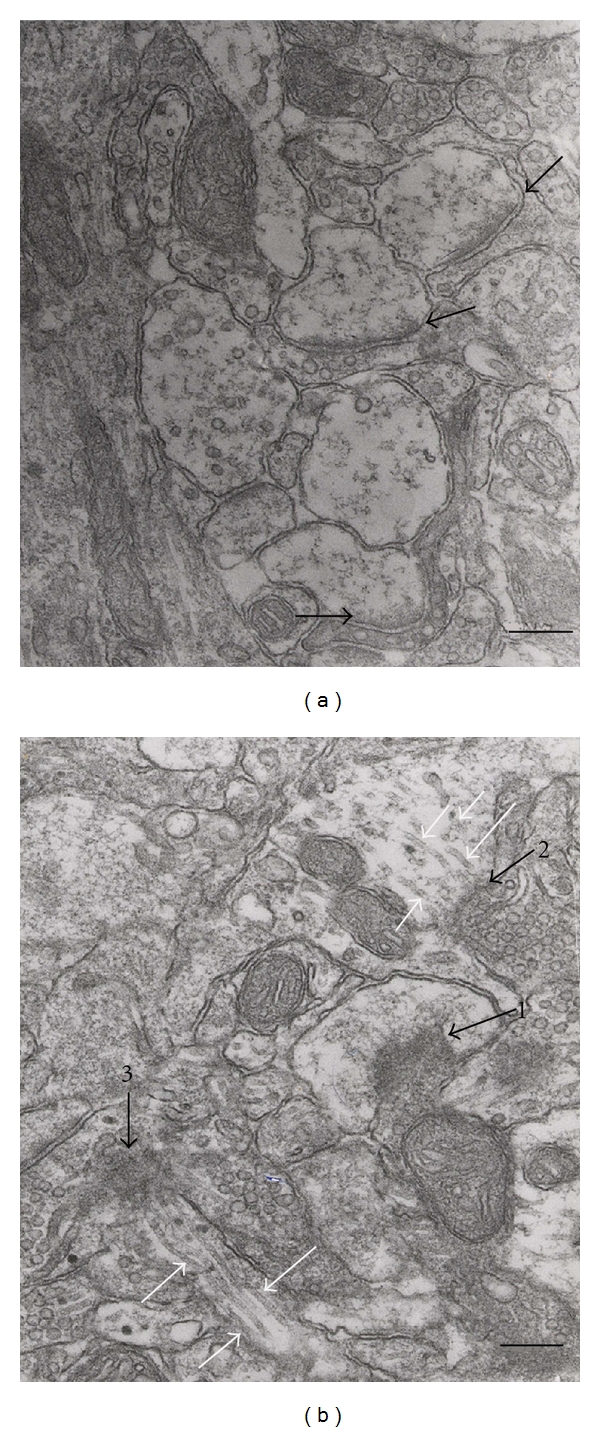
Redistribution of microtubules in dendritic spines after stimulation. There were little microtubules and thin postsynaptic densities (black arrows in (a)) in a nonstimulated slice (a). Many microtubules (white arrows in (b)) concentrated to three thickened PSDs (black arrows 1, 2, and 3 in (b)) in spines have emerged after stimulation (b). The planes of PSD1, 2, and 3 are perpendicular, oblique, and parallel to this electron microscopic section, respectively. Reprinted from Mitsuyama et al. (2008) [21] with permission. Bar, 150 nm.

Some previous studies have suggested that fewer microtubules were found in dendritic spines, but four recent reports [21–24] showed the capture of the plus ends of microtubules. Our previous studies [21], based on acute hippocampal slices fixed by a microtubule conserving process after LTP-inducing stimulation, showed that microtubules of the dendritic shaft ramified into spines (Figure 1) that were specific to the stimulated postsynaptic membranes (Figure 2). Because the microtubules originate from the centrosome which is localized next to the nucleus, these four reports strongly suggest a stimulation-dependent connection between the nucleus and the stimulated postsynaptic membrane by microtubules. This newly produced microtubule track, found primarily in the stimulated postsynaptic membrane, might be the route of bidirectional dendritic transportation of signals during LTP formation. Thus, the maintenance of intraspinal microtubules is crucial for memory storage in the normal brain.

In contrast, based on the research of AD mechanisms, there have been two main pathways, amyloid beta and tau. These two pathways of research originated from the facts that amyloid beta is the main component of senile plaques [25–27] and hyperphosphorylated tau is the main component of neurofibrillary tangles [28, 29]. Because it can be considered that the mechanisms of memory disturbance in AD can be explained by the impairment of normal memory mechanisms, the storage of amyloid beta or hyperphosphorylated tau focused our studies on the intraspinal microtubules.

A synaptic loss or spine loss has been described in patients with neurodegenerative disorders, such as AD [30, 31], and in their mouse models [32–35]. Such alterations are thought to be responsible for cognitive deficits before or even in the absence of neuronal loss, prior to understanding the true underlying mechanisms. Several pieces of evidence suggest that spine elongation may be caused by microtubule polymerization. This structural mechanism for spine elongation suggests, conversely, that synapse loss or spine loss observed in AD may be caused by the depolymerization of intraspinal microtubules. Amyloid activates GSK-3beta [36] and the activated GSK-3beta causes the abnormal hyperphosphorylation of tau and the depolymerization of axonal microtubules, resulting in the impairment of axonal transport [37]. Normal tau is primarily present in the axon, but hyperphosphorylated tau newly distributes to dendrites and sequesters microtubule-associated proteins (MAPs) such as normal tau, MAP1A/MAP1B, and MAP2 [38] and may cause the inhibition and disruption of intraspinal microtubules by losing the microtubule-preserving effect of MAPs. Nevertheless, it may be strongly suggested that amyloid beta may be a putative intraspinal microtubule-depolymerizer to induce spine loss and synaptic loss, resulting in the impairment of the bidirectional dendritic transports and finally the memory disturbance in AD.

## 2. Microtubules in the Dendritic Spines

Recently, there have been four reports that captured the plus ends of microtubules in dendritic spines [21–24]. In our report, based on acute hippocampal slices that were fixed by a microtubule conserving process after LTP-inducing stimulation, we showed that microtubules of the dendritic shaft ramified into spines (Figure 1) specifically into the stimulated postsynaptic membranes (Figure 2). This resulted in enlarged protrusions of dendritic spines [21]. Other reports using living cultured neurons showed that growing microtubule plus ends contain the microtubule tip-tracking protein EB3, enter into the spines, and modulate spine morphology [22–24]. Because the microtubules originate from the centrosome which is localized next to the nucleus, these four reports strongly suggest a stimulation-dependent connection between the nucleus and the stimulated postsynaptic membrane by the microtubules. It had been thought that microtubules in spines are less abundant. However, because they are very sensitive to disruption, it can be supposed that intraspinal microtubules had been depolymerized during conventional fixation. They were clearly delineated in the recent studies by using the microtubule-conserving fixation or the observation for living neurons.

## 3. Intraspinal Microtubules: They May Be Unstable and May Be Essential for Memory Storage

A number of studies have suggested that the expression in the late stages of LTP may require protein synthesis and gene expression [15, 16]. It has become commonly accepted that the trafficking of many plasticity-promoting molecules to the postsynaptic membranes is essential for memory storage [7–14], but the precise motor mechanism has not yet been discovered [39].

**Figure 3:**
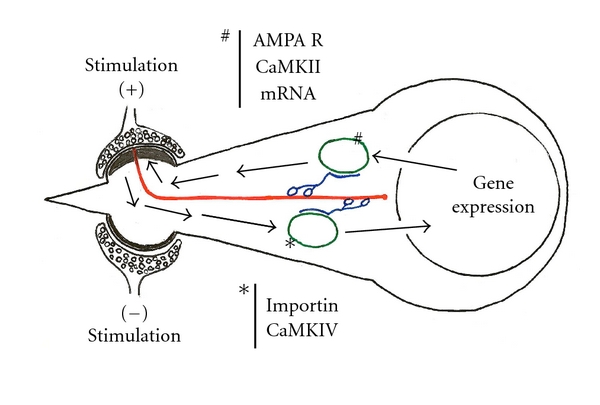
Memory storage. Hypothesis of “endless memory amplifying circuit” led by the intraspinal microtubules after LTP stimulation. Red: microtubules.

A novel mechanism based on microtubule dynamics can be proposed. The newly produced microtubules are localized only to the stimulated postsynaptic spine and that might be the route of the bidirectional dendritic transportation of signals during LTP formation. This led us to hypothesize the “endless memory amplifying circuit” (Figure 3), proposing that retrograde gene expression-promoting molecules, such as CaMKIV, are translocated from postsynaptic membrane to the cell body, enter into the nucleus, and activate transcription factors; anterograde gene products such as AMPA receptors [39] and CaMKII may be retranslocated only to the stimulated postsynaptic membrane along microtubules, as we have proposed previously [40].

## 4. Anterograde Signal Translocation and Newly Formed Microtubule Tracks

In dendritic shafts, it is commonly believed that these anterogradely translocated signals may be moved along with microtubules by microtubule-based motors. It has been suggested that myosin may function to transport these signals locally within the spine, because the actin filaments are the main cytoskeletal elements in the spine [41]. From the report that the EBI-melanophilin-myosin Va + TIP complex may have a role in focusing the transfer of melanosomes from the microtubule to actin at the plus end of microtubules [42], it has been suggested that track switching might occur at the transition of a dendritic shaft to a spine. However, there was another major finding using electron microscopy with conventional fixation that suggested that there were a small number of microtubules in either stimulated or nonstimulated spines. Recently, four reports indicated that new microtubules were produced by LTP-inducing stimulation from the dendritic shaft to the stimulated postsynaptic membrane, suggesting a mechanism for anterograde translocation of molecules along microtubules, even at the level of the spine. Therefore, it may be more reasonable to suppose that microtubule-plus-end capturing occurs at the LTP-induced stimulated postsynaptic membrane, similar to the leading edge in migrating cells [43].

## 5. Retrograde Signal Translocation and Newly Formed Microtubule Track

For signal transmission from the synapse to the nucleus, there are several pathways to be considered in CA1 neurons, such as dendritic action potential firing, calcium wave propagation, or the translocation of protein. To form reliable memories using only the plasticity changes (LTP) of individual synapses in the CA1 region, it may be crucial that the difference of plasticity between the stimulated and nonstimulated synapses is amplified. From this point of view, the transmission using action potentials and calcium waves may not be adequate, because many adjacent nonstimulated synapses will also be stimulated. The translocation of proteins to transmit signals from stimulated synapses to the nucleus, the retrograde signal translocation, appears as a more appropriate, selective mechanism to form memory. Hence, previous studies have reported that the suggested nuclear transport carrier importin was translocated from peripheral neurites to the nucleus in cultured *Aplysia* sensory neurons and in rat cultured hippocampal neurons by glutamatergic stimulation [44, 45].

 Because it has been reported that importin alpha transports CaMKIV into the nucleus without utilizing importin beta [46], it may be considered that CaMKIV is translocated conjugated with importin from the stimulated synapse to the nucleus and, consequently, activate the nuclear CREB protein. Accounting for the distance and velocity of importin translocation in neurites, importin may be translocated along microtubules by the microtubule motors. Thus, the newly produced microtubule track between the cell body and the stimulated postsynaptic membrane is the best candidate to be the track of this retrograde signal translocation.

How is the memory formed after the signals were transmitted to the nucleus and the nuclear transcription level is increased? The difference of plasticity between the stimulated and nonstimulated synapses may not be amplified just by the increase of the transcription level in CA1 neurons. It may be reasonable to think of mechanisms for the gene products that will promote plasticity, to be retranslocated only to the stimulated synapses. The newly produced microtubules between the cell body and stimulated postsynaptic membranes can guarantee this retransport system exclusively reaches the stimulated synapses.

## 6. Polymerization of Intraspinal Microtubules May Cause Spine Elongation

**Figure 4:**
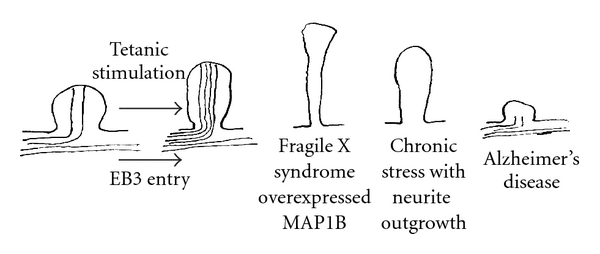
Spine shape change. Hypothesis for the induction of synapse loss and spine loss in Alzheimer's disease due to the depolymerization of intraspinal microtubules.

As previously noted, LTP-producing stimulation results in the ramification of dendritic shaft microtubules into the stimulated spines, resulting in spine enlargement [21]. Other evidence suggests that spine elongation may be caused by microtubule polymerization. First, the EB3-GFP entry into spines accompanies spine enlargement [24]. Furthermore, MAP1B is overexpressed in Fragile X syndrome, in which spines are elongated. The Fragile X protein is a mRNA-binding protein. It is likely that the mutation of this protein causes the impairment of mRNA transport to the local spine, resulting in low local protein synthesis. It is likely that MAP1B is overexpressed by a negative feedback mechanism, because the mRNA is translocated along microtubules [47]. Chronic stress causes neurite outgrowth and spine elongation in the lateral nucleus of the amygdala [48]. In addition, the polymerization of microtubules causes neurite outgrowth [49], and the microtubules-depolymerizing agent caused neurite retraction [50]. These findings are consistent with the proposition that spine elongation is caused by microtubule polymerization (Figure 4).

## 7. Depolymerization of Intraspinal Microtubules May Cause Spine Loss and Synaptic Loss

This structural mechanism for spine elongation suggests, conversely, that the synapse loss or spine loss observed in AD may be caused by the depolymerization of intraspinal microtubules. Many dendritic shaft's microtubules were present before the stimulation, even using conventional fixation. This indicates that the shaft's microtubules are very stable. Many intraspinal microtubules emerged after the stimulation only when the microtubule-preserving methods were adopted during the fixation, indicating their instability (Figures 1 and 2) [21]. To maintain the newly produced unstable microtubules in dendritic spines that may be essential to form memory, the dendritic spines probably enlarge by the pushing force of microtubules extended into the postsynaptic membranes, which were polymerized by the postsynaptic stimulation (Figure 4).

## 8. Amyloid Beta Is Intra-Axonal Microtubule Depolymerizer via Tau Phosphorylation

MAPs are present as regularly spaced projections on microtubules and consisted of tau, MAP1A/MAP1B, and MAP2. MAPs have microtubule-preserving effects, and they lose this effect when MAPs become phosphorylated [51]. Normal tau is primarily found in axons and, to a lesser extent, in dendrites. Amyloid activates GSK-3beta [36], and activated GSK-3beta causes the abnormal hyperphosphorylation of tau and the depolymerization of axonal microtubules, resulting in the impairment of axonal transport. To date, it may be the main hypothesis for the impairment of axonal transport as being the main cause of AD [37].

## 9. Amyloid Beta May Be an Intraspinal Microtubule Depolymerizer

Normal tau is mainly present in the axon, but hyperphosphorylated tau is likely detached from the axonal microtubules and redistributed to the dendrites. There it sequesters dendritic MAPs, such as normal tau, MAP1A/MAP1B, and MAP2 [38] and probably causes inhibition and disruption of intraspinal microtubules. It has also been reported that mitogen-activated protein kinase (MAPK) is activated when amyloid beta is conjugated with surface receptors of neurons [52]. Nevertheless, it may be strongly suggested that amyloid beta is a putative intraspinal microtubule depolymerizer to induce spine loss and synaptic loss, resulting in the impairment of the “endless memory amplifying circuit” [40] and finally to the memory disturbance in AD [53–55]. The fact that amyloid beta caused dendritic and axonal retraction followed by neuronal death [56] strongly supports this idea.

## 10. Intraspinal Microtubules May Be Critical for Neuronal Death

There are many different studies that examine the mechanism of neuronal death in AD, but intraspinal microtubules might be contributing to neuronal death. In the depolymerization of intraspinal microtubules, the retrograde signal translocation may be impaired, resulting in the decrease of nuclear stimulation. This reduced transcription level may severely affect the neuronal cell survival.

## 11. Conclusions

Synapse loss and spine loss have been well described in AD. Several pieces of evidence have suggested that spine elongation may be caused by microtubule polymerization. LTP-producing stimulation results in the ramification of dendritic shaft microtubules into stimulated spines, causing spine enlargement. The entry of microtubule plus ends into spines accompanies spine enlargement. Further, MAP-1B is overexpressed in Fragile X syndrome, in which spines are significantly elongated. Chronic stress causes neurite outgrowth and spine elongation. Polymerization of microtubules causes neurite outgrowth, and a microtubule-depolymerizing agent causes neurite retraction, both of which are consistent with the hypothesis that spine elongation is caused by microtubule polymerization. This spine elongation may be caused by the pushing of microtubules against the postsynaptic membrane like a tent pole due to the polymerization of microtubules by postsynaptic stimulation. This structural mechanism for spine elongation suggests, conversely, that the synapse loss and spine loss observed in AD may be caused by the depolymerization of intraspinal microtubules. It has been reported that amyloid activates GSK-3beta and that activated GSK-3beta causes the abnormal hyperphosphorylation of tau and depolymerization of axonal microtubules, resulting in the impairment of axonal transport. Normal tau is primarily present in the axon, but hyperphosphorylated tau newly distributes to the dendrites and sequesters normal tau, MAP1A/MAP1B, and MAP2 and may disrupt intraspinal microtubules by eliminating the microtubule-preserving effect of MAPs. Therefore, amyloid beta is strongly suspected to be a putative intraspinal microtubule depolymerizer that induces spine shortening, spine loss, synapse loss, dysfunction of plasticity-related molecules, and, ultimately, memory disturbances in AD.
